# Effect of Organic Substrates on the Photocatalytic Reduction of Cr(VI) by Porous Hollow Ga_2_O_3_ Nanoparticles

**DOI:** 10.3390/nano8040263

**Published:** 2018-04-22

**Authors:** Jin Liu, Huihui Gan, Hongzhang Wu, Xinlei Zhang, Jun Zhang, Lili Li, Zhenling Wang

**Affiliations:** 1 Henan Key Laboratory of Rare Earth Functional Materials, International Joint Research Laboratory for Biomedical Nanomaterials of Henan, The Key Laboratory of Rare Earth Functional Materials and Applications, Zhoukou Normal University, Zhoukou 466001, China; jinliu@zknu.edu.cn (J.L.); hongzhang_wu@zknu.edu.cn (H.W.); zhangxinlei@zknu.edu.cn (X.Z.); 18238260132@163.com (J.Z.); 2 School of Civil Engineering and Architecture, Ningbo Institute of Technology, Zhejiang University, Ningbo 315100, China; hhgan@nit.zju.edu.cn; 3 School of Life Science and Agriculture, Zhoukou Normal University, Zhoukou 466001, China

**Keywords:** Ga_2_O_3_, porous, Cr(VI), organic pollutants

## Abstract

Porous hollow Ga_2_O_3_ nanoparticles were successfully synthesized by a hydrolysis method followed by calcination. The prepared samples were characterized by field emission scanning electron microscope, transmission electron microscope, thermogravimetry and differential scanning calorimetry, UV-vis diffuse reflectance spectra and Raman spectrum. The porous structure of Ga_2_O_3_ nanoparticles can enhance the light harvesting efficiency, and provide lots of channels for the diffusion of Cr(VI) and Cr(III). Photocatalytic reduction of Cr(VI), with different initial pH and degradation of several organic substrates by porous hollow Ga_2_O_3_ nanoparticles in single system and binary system, were investigated in detail. The reduction rate of Cr(VI) in the binary pollutant system is markedly faster than that in the single Cr(VI) system, because Cr(VI) mainly acts as photogenerated electron acceptor. In addition, the type and concentration of organic substrates have an important role in the photocatalytic reduction of Cr(VI).

## 1. Introduction

Heavy metal ions from wastewater have become the primary threat to the human environment with the development of industrial civilization [1,2,3,4]. Hexavalent chromium (Cr(VI)) is a typical heavy metal contaminant with high solubility and toxicity, which originates from various industrial processes such as electroplating, leather tanning, and paint manufacture [5]. A common method of treating Cr(VI) in wastewater is to convert it into low toxic Cr(III), which can be precipitated as Cr(OH)_3_ in neutral or alkaline solutions, and removed as a solid waste [6]. Recently, photocatalytic reduction of Cr(VI) to Cr(III) has been recognized as an efficient and economical form of technology [7,8,9,10,11]. Briefly, photocatalytic reduction of Cr(VI) is based on the photogenerated electrons in the conduction band of semiconductor when it is irradiated by UV/visible light having energy greater than the band gap energy of the semiconductor. In addition, organic and inorganic pollutants usually co-exist in industrial wastewater and natural aqueous environment, and no doubt the presence of organic pollutants in wastewater will greatly increase the difficulty of photocatalytic reduction of Cr(VI) [12,13,14].

During the photocatalytic process, the photocatalyst is the key factor, and it is necessary to design and fabricate efficient and stable photocatalysts. Ga_2_O_3_ is one of most popular photocatalysts used in the photocatalytic degradation of organic pollutants and reduction of CO_2_ [15,16,17,18], owing to its high activity and environmental friendliness. Its activity can be further enhanced through a proper synthetic strategy to obtain nanostructured materials, as the morphology, size and pore structure of materials can significantly influence their properties and applications [19,20,21]. As one promising field of research, porous hollow nanostructures have been investigated for a long time. Compared with bulk materials, porous hollow materials have higher porosity, larger specific surface areas, and lots of active chemical sites, which could enhance light harvesting efficiency and provide lots of channels for the diffusion of pollutants, while also improving photocatalytic activity efficiently [22,23,24].

In this study, porous hollow Ga_2_O_3_ nanoparticles were prepared via a hydrolysis method followed by calcination. The effect of parameters including pH and concentration of metronidazole on the reduction rate of Cr(VI) by the porous hollow Ga_2_O_3_ nanoparticles was also studied. Meanwhile, the photocatalytic reduction of Cr(VI) was also systematically investigated in the absence and presence of organic substrates. To the best of our knowledge, this is the first report on the simultaneous treatment of organics and Cr(VI) using porous hollow Ga_2_O_3_.

## 2. Materials and Methods

### 2.1. Materials

Ga_2_O_3_ (99.999%), NaCO_3_ (AR, 99.8%), and NaOH (AR, 96%) were purchased from Shanghai Aladdin Bio-Chem Technology Co., Ltd. (Shanghai, China) and HCl (AR, 36–38%) were purchased from Sinopharm Chemical Reagent Co., Ltd. (Shanghai, China) Deionized water was used throughout the experiments.

### 2.2. Synthesis of Porous Hollow Ga_2_O_3_ Nanoparticles

Porous hollow Ga_2_O_3_ nanoparticles were synthesized via a thermal transformation of GaOOH precursor based on our previous study [25]. The NaGaO_2_ powders were prepared by heating a stoichiometric mixture of Na_2_CO_3_ and Ga_2_O_3_ at 850 °C for 12 h. The GaOOH precursor was prepared by a hydrolysis reaction of NaGaO_2_ colloidal solution. The NaGaO_2_ powders (1.0 g) was dispersed in deionized water (100 mL) to obtain a colloidal solution with ultrasonic oscillations. Then, 5 mol/L HCl solution was added to the NaGaO_2_ colloidal solution with magnetic stirring; the final pH value was kept at 9.0. The obtained white suspension was treated thermally at 80 °C for 12 h. The obtained GaOOH precursor was separated by centrifugation and dried at 70 °C for 10 h. The Ga_2_O_3_ was prepared by calcining GaOOH precursor with a programmed temperature (400 °C, 5 h and 700 °C, 1.5 h, 1 °C/min).

### 2.3. Characterization

The Raman spectrum was recorded using a Raman spectrometer (RM2000) (Renishaw, Gloucestershire, UK). Field emission scanning electron microscope (FESEM) images were obtained using a MERLIN scanning microscope at an accelerating voltage of 10 kV (ZEISS, Oberkochen, German). Scanning transmission electron microscopy (STEM), transmission electron microscope (TEM) and high-resolution transmission electron microscopy (HRTEM) images were obtained using a JEOL-2010 transmission electron microscope (JEOL Ltd., Kyoto, Japan) at an accelerating voltage of 200 kV. TEM is equipped with an energy-dispersive X-ray spectroscopy (EDS) analysis system. The quantitation method for Ga and O elements is Cliff Lorimer thin ratio section. Thermogravimetry and differential scanning calorimetry (TG-DSC) analysis was performed on a STA 6000 (Perkin Elmer, Waltham, MA, USA) instrument at a heating rate of 10 °C/min. UV-vis diffuse reflectance spectra (UV-vis DRS) were obtained by a UV-2600 UV-vis spectrophotometer (Shimadzu Corporation, Kyoto, Japan).

### 2.4. Photocatalytic Experiments

Photoreduction of Cr(VI) (K_2_Cr_2_O_7_) and photocatalytic degradation of rhodamine B (RhB), acid red 1 (AR1), methyl orange (MO) and metronidazole (MNZ) as well as their binary mixtures, were adopted to evaluate the photocatalytic activity of the as-synthesized Ga_2_O_3_ sample. The concentration of Cr(VI) and organics is the same in single and binary pollutants (Table 1). Typically, 20 mg of the Ga_2_O_3_ sample was added into a 50 mL Cr(VI) aqueous solution. The initial pH of the Cr(VI) solution was adjusted to 2–9 by adding HCl or NaOH. Prior to irradiation, the suspensions were magnetically stirred for 30 min to establish the adsorption-desorption equilibrium. The irradiation was performed with a 30 W UV light lamp (λ = 253.7 nm). At a given time interval, about 3 mL suspension was taken for further analysis after centrifugation. The concentration of organic pollutants, including RhB, AR1, MO and MNZ, were analyzed by UV-vis spectroscopy at 554, 505, 464 and 320 nm, respectively. Meanwhile, the concentration of Cr(VI) was analyzed by a 1,5-diphenylcarbazide spectrophotometric method with a spectrophotometer at 540 nm (GB 7466-87, Standards of China). The characteristic absorbance peaks of organic pollutants (rhodamine B, acid red 1, methyl orange, metronidazole) are different; their absorbances are different when the concentrations are same. In order to quickly measure the absorbance of organic pollutants by UV-vis spectroscopy, we created the proper concentrations.

## 3. Results and Discussion

### 3.1. Composition and Morphology

**Figure 1:**
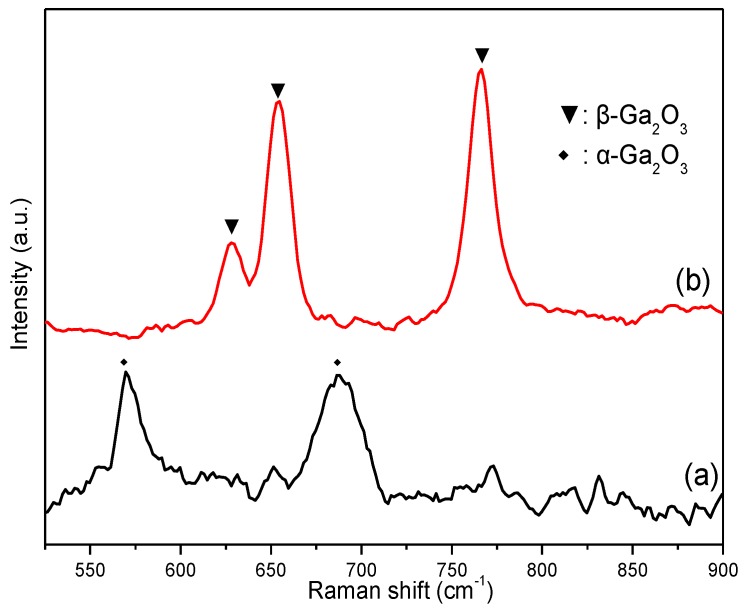
Raman spectra of Ga_2_O_3_ from the GaOOH precursor calcined at (**a**) 600 °C and (**b**) 700 °C.

Previous X-ray diffraction (XRD) results show that the phase composition of the GaOOH precursor and its calcined product are α-GaOOH (JCPDS No. 06-0180) and β-Ga_2_O_3_ (JCPDS No. 41-1103), respectively [25]. The average crystallite size of β-Ga_2_O_3_ sample is about 27.3 nm by the Scherrer equation. [26] The composition of Ga_2_O_3_ samples was further investigated by Raman spectra, owing to the greater sensitivity of Raman spectroscopy to the outer region of the solid samples than XRD [27]. The Raman spectra of Ga_2_O_3_ samples from the GaOOH precursor—calcined at 600 °C and 700 °C—are shown in Figure 1. The characteristic Raman bands of α-Ga_2_O_3_ and β-Ga_2_O_3_ are shown in Figure 1a,b, respectively; this is consistent with the reported results [28,29]. This result indicates that the α-Ga_2_O_3_ gradually transforms into β-Ga_2_O_3_ with the increase of calcination temperature; pure phase β-Ga_2_O_3_ is finally obtained at 700 °C.

**Figure 2:**
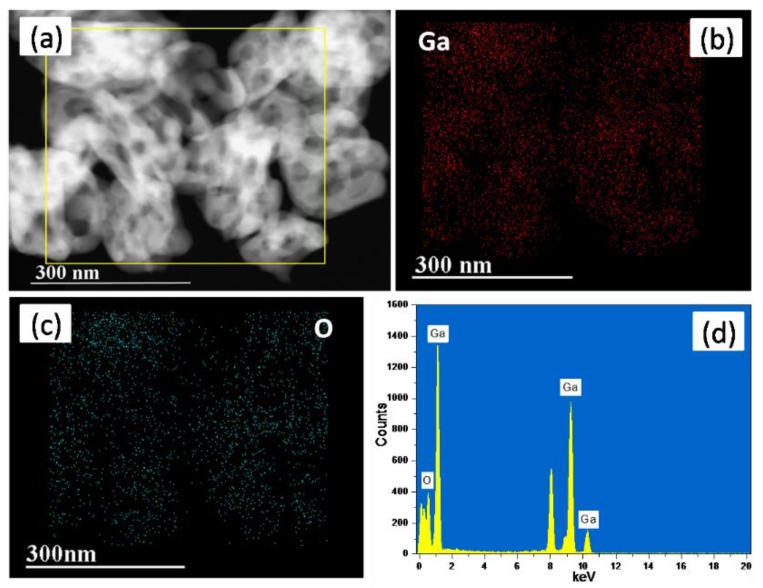
(**a**) Scanning transmission electron microscopy (STEM) image, (**b**,**c**) energy-dispersive X-ray spectroscopy (EDS) elemental mapping images and (**d**) EDS spectrum of Ga_2_O_3_.

The composition of porous hollow Ga_2_O_3_ nanoparticles was also analyzed by EDS elemental mapping images and EDS spectrum. As shown in Figure 2, it is clearly seen that Ga_2_O_3_ nanoparticles possess a porous structure, and that Ga and O elements are distributed homogenously in the Ga_2_O_3_ sample and their atomic ratio was close to 2:3, which further indicates that the synthesized sample is pure Ga_2_O_3_.

**Figure 3:**
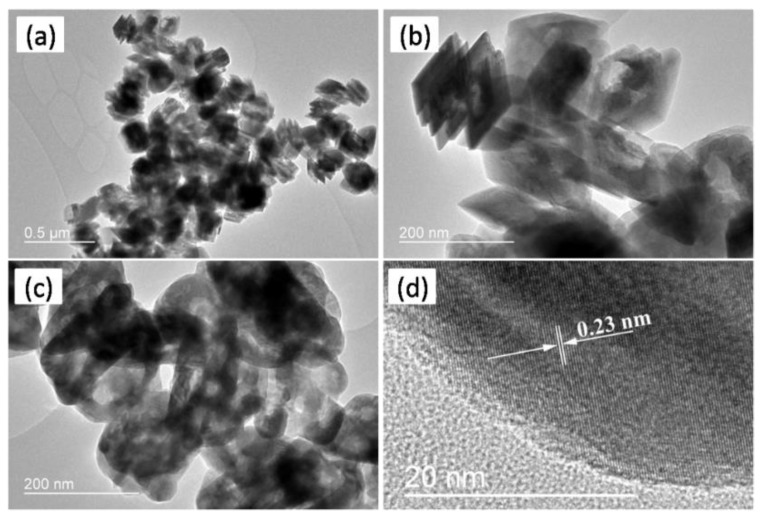
Transmission electron microscopy (TEM) and high-resolution transmission electron microscopy (HRTEM) images of (**a**,**b**) GaOOH and (**c**,**d**) Ga_2_O_3_.

**Figure 4:**
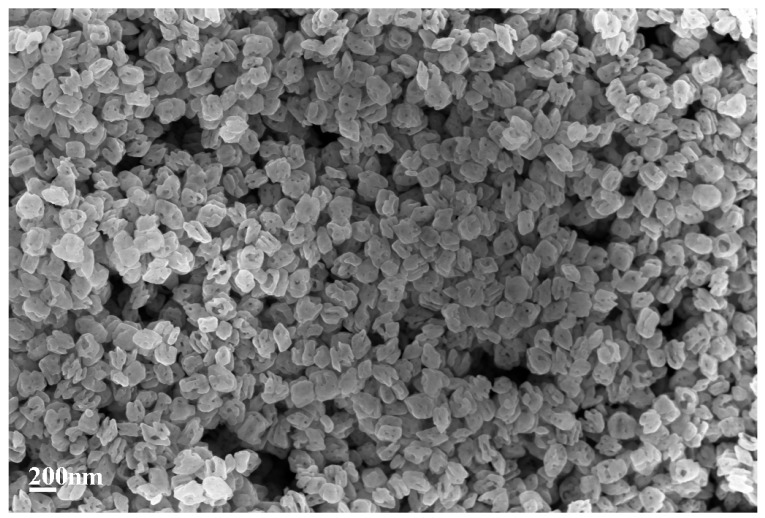
Scanning electron microscope (SEM) image of Ga_2_O_3_ nanoparticles.

**Figure 5:**
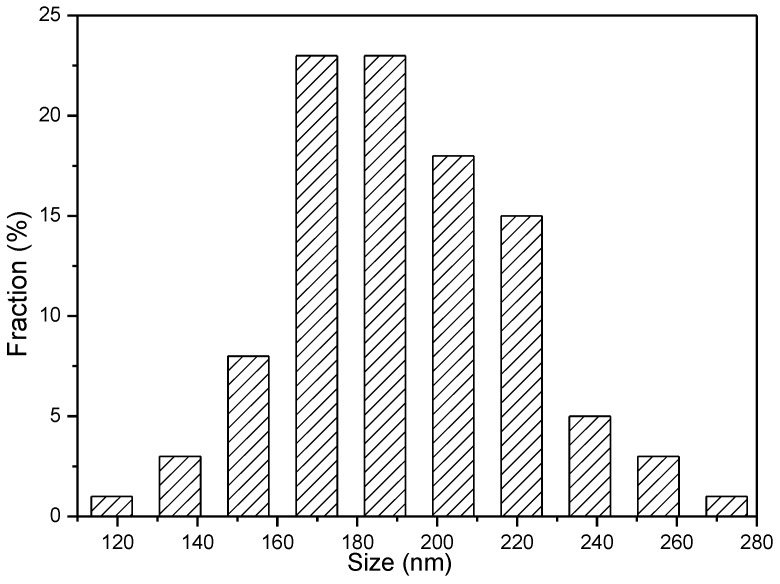
The size distribution of Ga_2_O_3_ nanoparticles.

The morphology and microstructure of GaOOH precursor and its calcined product Ga_2_O_3_ were investigated by TEM and SEM. As shown in Figure 3a,b, the GaOOH precursor presents monodisperse nanoplate-like structure. Compared with GaOOH (Figure 3a,b), Ga_2_O_3_ nanoparticles (Figure 3c) present porous hollow structures. Moreover, the clearly resolved lattice fringes with d spacing of 0.23 nm (distance between two arrow heads in Figure 3d) correspond to the ($\bar{3}$11) lattice planes of monoclinic β-Ga_2_O_3_, which is in good agreement with the XRD result. Figure 4 also shows that the Ga_2_O_3_ nanoparticles possess hollow structures [25]. The porous hollow structure of Ga_2_O_3_ nanoparticles is mainly ascribed to the thermal dehydration of the GaOOH precursor. These porous structure can enhance the light harvesting efficiency and provide lots of channels for the diffusion of Cr(VI) and Cr(III), resulting in the improvement of photocatalytic efficiency [30]. The size distribution of Ga_2_O_3_ in Figure 5 was evaluated from the SEM image (Figure 4) by measuring the diamenter of about 100 nanoparticles. It is clearly seen that the size of most Ga_2_O_3_ nanoparticles is 160–230 nm. 

### 3.2. Hermogravimetry and Differential Scanning Calorimetry TG-DSC Analysis

**Figure 6:**
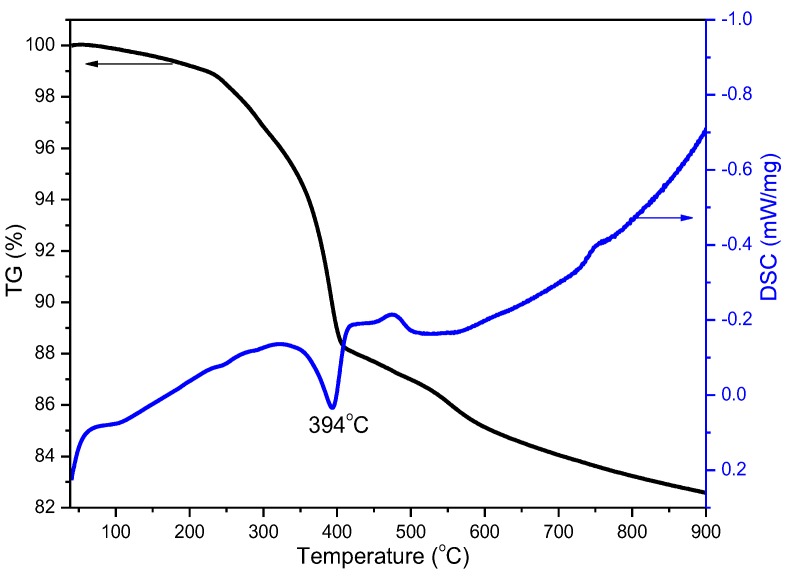
Hermogravimetry and Differential Scanning Calorimetry TG-DSC curve of the GaOOH precursor.

To understand the thermal conversion of the GaOOH precursor to Ga_2_O_3_, a TG-DSC measurement was performed. The TG-DSC measurement, performed from 40 to 900 °C for the GaOOH precursor, is shown in Figure 6. The major exothermic peak at about 394 °C was probably caused by the phase transformation of the sample from GaOOH to Ga_2_O_3_, as evidenced by a weight loss of 12% in the range of 40–400 °C in TG curve. A weight loss of 3% in the range of 400–600 °C, which is demarcated by weak endothermic peak in DSC curve, indicates the conversion of α-Ga_2_O_3_ to β-Ga_2_O_3_ above 600 °C [31]. The result is consistent with the Raman band of the GaOOH precursor calcined at 600 °C (Figure 1). The XRD, Raman spectra, and TG-DSC results indicate that the pure phase β-Ga_2_O_3_ can be obtained at 700 °C, and that α-Ga_2_O_3_ gradually transforms into β-Ga_2_O_3_ in the temperature range of 600–700 °C.

### 3.3. Photocatalytic Experiments

#### 3.3.1. Photocatalytic Reduction of Cr(VI)

**Figure 7:**
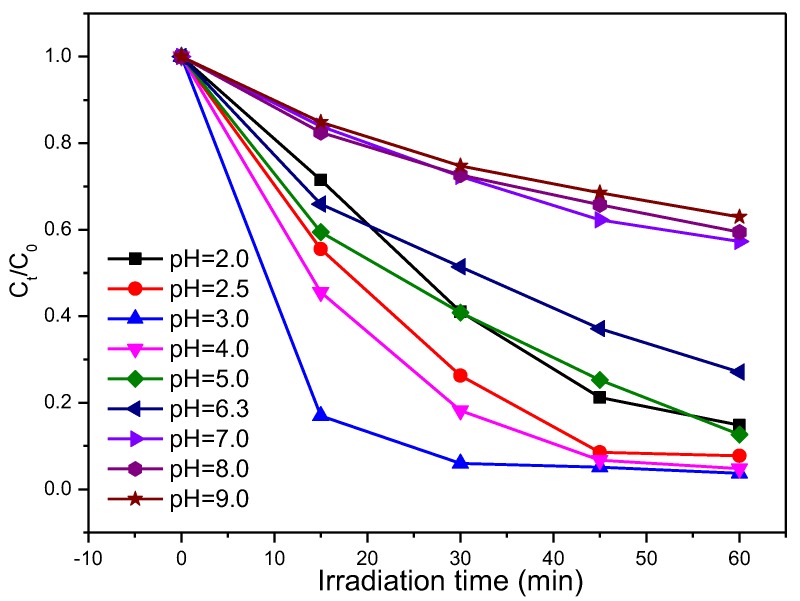
Photocatalytic reduction of Cr(VI) by Ga_2_O_3_ at different initial pH.

The pH of the solution is one of the most important parameters affecting the photocatalytic reduction of Cr(VI) on photocatalysts. The temporal concentration variation of Cr(VI) reduction by the porous hollow Ga_2_O_3_ nanoparticles at a pH range from 2.0 to 9.0 is shown in Figure 7. Obviously, the reduction of Cr(VI) is increased rapidly by decreasing the pH when the initial pH is in the range of 3–9. In general, the predominant form of Cr(VI) is Cr_2_O_7_^2^^−^ at a pH range of 2–6, while the major form was CrO_4_^2−^ at pH > 7 [32,33]. The photocatalytic reduction of Cr(VI) to Cr(III) consumes H^+^ in an acidic solution (Equation (1)), and produces OH^−^ in an alkaline solution (Equation (2)). At a low pH, the Ga_2_O_3_ nanoparticles are highly protonated and have a strong affinity toward the anion Cr_2_O_7_^2^^−^, and thus enhance the photocatalytic reduction of Cr(VI). However, the photocatalytic reduction of Cr(VI) is decreased when the initial pH is kept at 2–2.5, which may be attributed the dissolution of Ga_2_O_3_ nanoparticles. At a higher pH, the surface charge of the Ga_2_O_3_ nanoparticles will be less positively charged, or even negatively charged, which tends to electrostatically repel the anionic Cr(VI), and adsorb the cationic Cr(III) [34,35]. The electrostatical repulsion makes it more difficult for the anionic Cr(VI) to obtain the photogenerated electrons. Meanwhile, Cr(OH)_3_ precipitate will be formed at pH > 6, and occupies the active sites of Ga_2_O_3_ nanoparticles, leading to the decrease in the photocatalytic reduction of Cr(VI). Therefore, it is concluded that the photocatalytic reduction of Cr(VI) to Cr(III) is highly efficient at a suitable acidic condition, and is restrained at an alkaline condition.

(1) $${\mathrm{Cr}}_{2}O_{7}^{2-}+14H^{+}+6e^{-}\to 2{\mathrm{Cr}}^{3+}+7H_{2}O$$

(2) $$\mathrm{Cr}{O_{4}}^{2-}+4H_{2}O+3e^{-}\to \mathrm{Cr}{\left(\mathrm{OH}\right)}_{3}+5{\mathrm{OH}}^{-}$$

#### 3.3.2. Photocatalytic Degradation of Organic Pollutants

**Figure 8:**
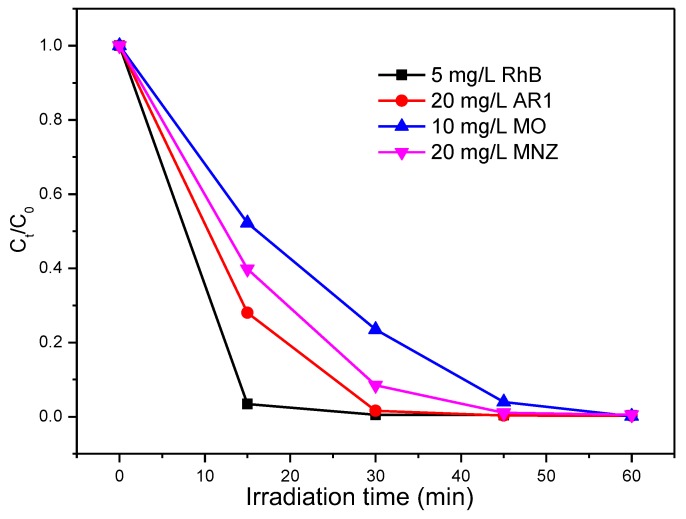
Photocatalytic degradation of single pollutant by Ga_2_O_3_.

Besides the photocatalytic reduction of Cr(VI), typical organic pollutants such as RhB, AR1, MO and MNZ were also used to evaluate the photocatalytic activity of porous hollow Ga_2_O_3_ nanoparticles. As shown in Figure 8, these four pollutants can be effectively degraded by the Ga_2_O_3_ nanoparticles in 60 min when they are in the single pollutant system (Table 1). The results indicate that the porous hollow Ga_2_O_3_ is a promising photocatalyst in water treatment.

#### 3.3.3. Simultaneous Treatment of Cr(VI) and Organic Pollutants

**Figure 9:**
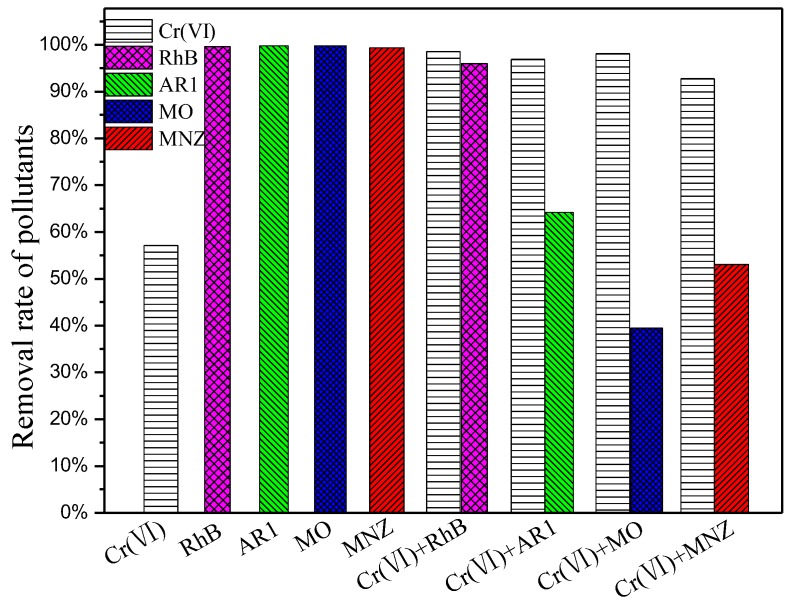
Photocatalytic treatment of single and binary pollutant system by Ga_2_O_3_.

Cr(VI) is often discharged together with hazardous organics from industrial wastewater. To further study the photocatalytic activity of porous hollow Ga_2_O_3_ nanoparticles, several binary pollutants were simulated using organics as the additional substrates. As shown in Figure 9, the reduction rate of Cr(VI) in the binary pollutants is markedly faster than that of the single Cr(VI). Cr(VI) mainly acts as photogenerated electron acceptor. However, the degradation rate of the organic pollutants in the binary pollutant system is lower than that of the corresponding single pollutant system. Based on our previous study, the photogenerated electrons play an important role in the degradation of organic pollutants by Ga_2_O_3_. The photogenerated electrons in the conduction band of Ga_2_O_3_ are assumed by Cr(VI), reducing the degradation rate of organic pollutants. The photocatalytic stability of the prepared Ga_2_O_3_ for the treatment of pollutants has been investigated by the recycling experiments. However, the photocatalytic reduction rate of Cr(VI) is 67% after two cycling runs. In order to activate the recycled Ga_2_O_3_ photocatalyst, an ultrasound treatment is used; a photocatalytic reduction rate of Cr(VI) is able to maintain 81%, under the same conditions. However, the photocatalytic reduction rate of Cr(VI) is only 44%, even after four cycling runs with the ultrasound treatment. The photocatalytic degradation rate of organic pollutants (RhB, AR1, MO, MNZ) is stable, even after five cycling runs.

#### 3.3.4. Effect of Substrate Concentration on Photocatalytic Reduction of Cr(VI)

**Figure 10:**
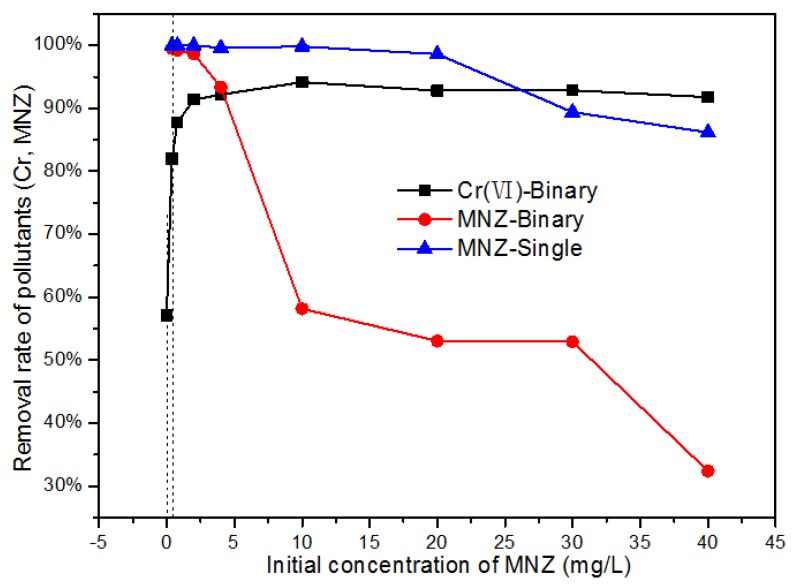
Photocatalytic treatment of Cr(VI) and MNZ in single and binary pollutant system.

To further assess the effect of organic pollutants as substrates on the photocatalytic reduction of Cr(VI), several different concentrations of MNZ in the Cr(VI)/MNZ binary pollutants were investigated. As shown in Figure 10, only 57% of Cr(VI) is photocatalytically reduced in the absence of MNZ after 60 min, and the reduction of Cr(VI) is increased striking when MNZ is added into the system. By increasing the concentration of MNZ to 10 mg/L, 94% of Cr(VI) is reduced. The reason may be that the presence of MNZ can consume the photogenerated holes in photocatalyst, and more photogenerated electrons are captured by Cr(VI), improving the photocatalytic reduction of Cr(VI). The reduction rate of Cr(VI) is not markedly changed when the concentration of MNZ is further increased.

## 4. Conclusions

In summary, porous hollow Ga_2_O_3_ nanoparticles were successfully synthesized by a hydrolysis method followed by calcination. It was demonstrated that the Ga_2_O_3_ photocatalyst is effective for the treatment of Cr(VI) and organic pollutants—as well as a mixture of them. The photocatalytic removal rate of Cr(VI) is highest when the initial pH of Cr(VI) is 3.0. The presence of organic pollutants in the reaction system improves the photocatalytic reduction of Cr(VI) by acting as a holes scavenger, leading to better charge carrier separation. The results broaden the range of approaches for the treatment of practical wastewater.

## Figures and Tables

**Table 1:** The concentration of Cr(VI) and organics in single and binary pollutant system.

Name	Single Pollutant System, mg/L	Binary Pollutant System, mg/L			
		RhB + Cr(VI)	AR1 + Cr(VI)	MO + Cr(VI)	MNZ + Cr(VI)
Cr(VI)	2.5	2.5	2.5	2.5	2.5
RhB	5	5	×	×	×
AR1	20	×	20	×	×
MO	10	×	×	10	×
MNZ	20	×	×	×	20
